# Meta-analysis data concerning popularity, theory of mind and interaction in experiments

**DOI:** 10.1016/j.dib.2019.104890

**Published:** 2019-11-30

**Authors:** Akihito Imai

**Affiliations:** Kobe University, Japan

**Keywords:** Interaction, Meta-analysis, Popularity, Theory of mind

## Abstract

This article describes data of effect sizes in studies on an association between theory of mind (ToM) and popularity. The data included 1946 children from 17 studies (22 effect sizes). The data are suitable for and were subjected to meta-regression to compare effect sizes of an interaction group (ToM was assessed in person) with that of non-interaction (ToM was assessed by computer).

**Table:** Specifications Table

Subject	Developmental and Educational Psychology
Specific subject area	Theory of mind
Type of data	Table
How data were acquired	Data was acquired from the published articles.
Data format	Raw and Analyzed
Parameters for data collection	Publication year, age, gender ratio, Pearson correlation coefficient between theory of mind and popularity, and assessment type (by computer and in person) were extracted from the studies included.
Description of data collection	Data were extracted from peer-reviewed journal articles, according to inclusion criteria. The electronic databases were searched for relevant articles. Citation search was also conducted.
Data source location	Kobe, Japan
Data accessibility	Data is within this article and repository. Repository name: Open Science Framework Data identification number: 82auq Direct URL to data: https://osf.io/82auq/

**Table:** 

**Value of the Data** • Data cover 1946 children from 17 studies (22 effect sizes), which allow for a reinvestigation of theory of mind. • Data are suitable for meta-analysis to review the relationships between variables. • Data may be used to increase awareness about overlooked effect sizes as well as can be used to compare with new results.

## 1 Data

The dataset contains data concerning popularity, theory of mind and interaction in experiments obtained from previous studies and were deposited at Open Science Framework. Data were extracted from peer-reviewed journal articles published until March 2017. According to criteria (see 2.1. Design, materials, and methods), 17 studies (22 effect sizes) were identified. Detailed characteristics were coded for each study and presented in Table 1. As shown, a total of 1946 children were included. Pearson correlation coefficient or *r* between theory of mind (ToM) and popularity varied from −0.06 to 0.49. Gender ratio varied from 0.42 to 0.58. Four effect sizes were assessed by computer (non-interaction) and 18 were assessed in person (interaction).

**Table 1:** Characteristics of studies included in the meta-regression.

Study	*N*	*r*	Girl ratio	Interaction	Assessment by	Note
Banerjee & Watling (2005) year 1 cohort [1]	113	0.07	0.52	No	a multimedia computer interface	synthesized (.08, .04, .09)
Banerjee & Watling (2005) year 4 cohort [1]	195	0.14	0.43	No	a multimedia computer interface	synthesized (.20, .09, .23)
Banerjee et al. (2011) younger group [2]	72	0.19	0.57	No	a multimedia computer interface	synthesized (.07, .16, .19, .05, .29, .39)
Banerjee et al. (2011) older group [2]	138	0.11	0.43	No	a multimedia computer interface	synthesized (.09, .23, .02, .11)
Braza et al. (2009) [3]	98	0.21	0.56	Yes	qualified, trained researchers	
Caputi et al. (2012) [4]	70	0.05	0.44	Yes	the experimenter	synthesized (.02, .04, .06, .07), time 2, 3
Cassidy et al. (2003) [5]	67	0.22	0.52	Yes	two female experimenters	synthesized (.09, .34)
Dockett & Degotardi (1997) [6]	24	0.46	0.54	Yes	the researcher	
Fink et al. (2014) [7]	114	0.30	0.49	Yes	the experimenter	synthesized (.35, .25), time 1, 2
Fink et al. (2015) [8]	114	0.35	0.49	Yes	interviewd individually	time 1
Flynn & Whiten (2012) [9]	88	0.34	0.58	Yes	the experimenter	
Hoglund et al. (2008) grade 2 [10]	114	0.15	0.50	Yes	the first author, research assistant helpers	
Kuhnert et al. (2017) [11]	114	0.12	0.49	Yes	interviewd individually	synthesized (.28, −.08), time 1, 2
Mizokawa & Koyasu (2011) [12]	102	0.19	0.55	Yes	the experimenter, the recorder	synthesized (.15, .32, .13, .15)
Morino (2005) junior class [13]	43	−0.06	0.51	Yes	the experimenter	
Morino (2005) middle class [13]	47	0.24	0.51	Yes	the experimenter	
Morino (2005) senior class [13]	47	0.40	0.49	Yes	the experimenter	
Peterson & Siegal (2002) [14]	109	0.32	0.40	Yes	the tester, the experimenter	synthesized (.31, .33)
Slaughter et al. (2002) study 1 [15]	78	0.27	0.47	Yes	a female experimenter	
Slaughter et al. (2002) study 2 [15]	87	0.11	0.47	Yes	a female experimenter	
Spence (1987) [16]	60	0.49	0.47	Yes	one of two research assistants	
Watson et al. (1999) study 2 [17]	52	0.41	0.42	Yes	the experimenter	

## 2 Experimental design, materials, and methods

### 2.1 Design, materials, and methods

The electronic databases, Psych INFO and Google Scholar were searched for relevant articles in March 2017. Search terms were as follows: “popularity,” “sociometric,” “peer acceptance,” “peer likability,” “peer rejection,” “peer status,” “peer evaluation,” “peer nomination,” “peer relations,” “ToM,” “mindreading,” “mentalizing,” “false belief,” “mental representations,” “mind understanding,” and “mental states” as used in a previous meta-analysis [18]. The Japanese database, J-STAGE, was also used to collect articles in Japanese. Citation search was also conducted.

The following inclusion criteria were used (criteria (i) to (iii) followed previous meta-analysis [18]): (i) Only healthy preschool or school-aged children under 10 years could participate; (ii) ToM had to be assessed by more than one of false-belief understanding, hidden emotion, affective perspective-taking, or faux pas tasks; (iii) Sociometric or perceived popularity had to be assessed by a peer or a teacher; (iv) Effect size(s), *N* and gender ratio must be reported or convertible. An association between ToM and popularity was evaluated by Pearson correlation coefficient or *r*. If multiple coefficients were reported in a study, these were synthesized into one coefficient unless a study has multiple age groups; (v) Measures of ToM have to be identifiable (computer or person). Coding is organized based on whether ToM was assessed by computer or in person such as researcher and experimenter. Studies assessing ToM by computer are categorized as a non-interaction group and those assessing ToM in person are categorized as an interaction group; (vi) Peer-reviewed articles were from publication in either English or Japanese.

### 2.2 Meta-analysis

Random-effects meta-regression was employed. The statistical software Stata 15.0 was used for all data analysis. The meta-regression algorithm was implemented by using version 2.6.1 of the *metareg* command [19]. Publication bias was assessed by using version 4.1.0 of the *metabias* command [20].

The result of meta-regression, without fitting any covariates, showed that effect size was associated with interaction in the experiments, β = 0.134 (95%CI 0.006 - 0.262), *p* = .041, and between-study heterogeneity (*I*^2^_res_) was 27.68%. After controlling for gender, the result was consistent, β = 0.137 (95%CI 0.003 - 0.272), *p* = .046, *I*^2^_res_ = 30.82%.

To overcome the small sample size which increases the chance of a false-positive (type I) error, permutation analysis was implemented. The result was marginally significant (*p* = .083). The result of Egger's test showed that publication bias was non-significant (*p* = .735).

## Conflict of interest

The authors declare that they have no known competing financial interests or personal relationships that could have appeared to influence the work reported in this paper.
